# Introduction of early health technology assessment for innovators, clinicians, and funders

**DOI:** 10.1038/s44401-026-00102-2

**Published:** 2026-06-19

**Authors:** Teerawat Wiwatpanit, Yot Teerawattananon, Yi Wang

**Affiliations:** 1 Health Intervention and Technology Assessment Program Foundation , Nonthaburi, Thailand; 2 Saw Swee Hock School of Public Health, National University of Singapore and National University Health System, Singapore, Singapore

**Keywords:** Business and industry, Health care, Mathematics and computing, Medical research

## Abstract

Health technology faces rising costs, system complexities, and gaps between development and coverage policies, limiting translation into practice. Traditional health technology assessment (HTA) evaluates technologies at late stages, providing little support if a technology fails to secure reimbursement. Early HTA addresses this by assessing technologies during research and development stages, helping innovators prioritise disease areas, define target product profiles, and establish value propositions. Early HTA could reduce development risks and uncertainties and inform development choices that could improve cost-effectiveness and enhance long-term viability, adaptability, and adoptability in the healthcare system.

## Introduction

Health and biomedical innovators face increasing demands for new technologies. However, despite global advances in healthcare, the rising costs of health technologies, including interventions such as tests, devices, medicines, vaccines, procedures, programmes, and system-level tools, pose significant barriers to their implementation^1–4^. This phenomenon is more prominent in the era of universal health coverage when most governments commit to offering affordable healthcare services to all those in need^5–8^. The complexity of the healthcare sector, with its many stakeholders and agendas, often leads to a misalignment between health technology development, primarily driven by scientists, and health technology coverage policies influenced by the government, healthcare professionals, and patients. In addition, there are also challenges to implement new technologies beyond the research and development (R&D) stages, including lengthy process of deploying advanced medical technologies and insufficient reimbursement^9,10^. Given these challenges in translating research into real-world applications, there is a need to reform the health technology market, specifically to bridge the disconnection between innovation development and the public reimbursement^11^.

The process from ideas to clinical application is lengthy and expensive, with about 17-year gap between research evidence and clinical practice^12,13^. In the US alone, the National Institute of Health invested most of its $45 billion budget in medical research in 2023^14^. The funding needed for developing a medical device with moderate risk can be up to $30 million^3^. However, 30–90% of health technologies fail to enter the healthcare system due to the lack of target validations and optimisation during the R&D stages^15,16^. The high R&D investment leads to the delegation of costs to patients and payers, making innovations even less affordable and feasible for inclusion in public reimbursement schemes. Medical innovators need measures to assess technology viability and value, taking proactive steps to address potential challenges and ensure a successful translation of their medical research from bench to bedside^17^. In light of this, early health technology assessment (early HTA) was proposed to guide health technology development. However, the awareness and usage of early HTA remain suboptimal among innovators and clinicians^18^. Challenges exist for applying early HTA on the ground^19,20^. There are also misperceptions and misuse of early HTA.

To fill in the abovementioned gap and facilitate the adoption of early HTA among funders, innovators, clinicians, and decision-makers, this perspective article draws on research and capacity-building activities in early HTA and related initiatives in Thailand and Singapore. It aims to introduce early HTA with a focus on practical and effective methods for evaluating and assessing the value of health technologies across the product life cycle. By further integrating a targeted review of the relevant early HTA and HTA literature, this perspective article provides an evidence-informed perspective on current practices, common misconceptions, and key challenges in conducting early HTA.

## What is traditional HTA?

**Fig. 1:**
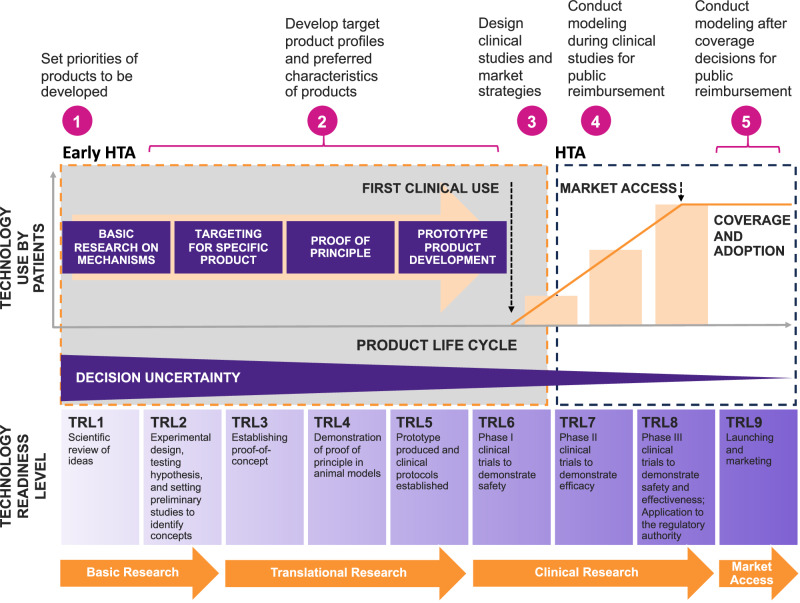
Medical product life cycle and technology readiness level (TRL). Early health technology assessment (early HTA) takes part in the development of a health technology at the early stages: basic research, translational research, clinical research, and market access^45^^,59^. Early HTA prioritises disease areas, identifies desirable characteristics of the technology and lays fundamentals for clinical trials and market strategies through early dialogues with stakeholders and policymakers, and early economic modelling. Image further developed based on findings from^46^.

Traditional HTA, a multidisciplinary process, employs evidence-based and systematic approaches to evaluate the effectiveness, values, and impacts of health technology primarily at the post-market authorisation stage^21–23^ (Fig. 1). Although HTA can occur at the pre-market stage, this typically does not correspond to “early HTA” conducted at the R&D stage. HTA offers insights that enable informed decision-making, resource allocation, and investment to optimise outcomes valued by each stakeholder group or the public^22,24,25^. Traditional HTA usually occurs once sufficient evidence, like efficacy/effectiveness or market pricing, has been gathered. It often deals with assessing economic impacts, from societal, healthcare-system, or payer’s perspective, as well as socioeconomic, organisational, clinical, and ethical impacts of the selected health technologies, including patient engagement activities to ensure patient perspectives are incorporated^26^. However, when the evidence is immature or insufficient, traditional HTA could still be conducted to inform conditional reimbursement and managed entry agreements, and subsequently reassessment may be required. HTA also informs who is responsible for the cost of implementing these innovations in clinical practices. When policymakers use traditional HTA to assess the value of these innovations and the innovations do not meet the evaluation criteria, these innovations often fail to be included in health benefit packages^27^.

Furthermore, the traditional HTA does not factor in the challenge and uncertainty of developing the innovation itself at the R&D stage. Many health technologies, though with promising clinical results, enter the health market but later fail to get implemented in the healthcare system because they lack desirable product profiles to compete with existing products^28,29^. While traditional HTA serves as a tool to ensure the successful adoption of new health technologies, it sometimes can pose barriers that hinder clinically impactful innovations from entering the healthcare system.

## What is early HTA?

Early HTA plays an important role in facilitating the development of emerging health technologies by gathering evidence from multiple perspectives during the R&D stage. The use of early-stage economic evaluation (EE) for medical technology dates back to around the 1990s^20^, initially focusing on drug development^30–32^ before expanding toward medical devices and interventions^33–36^. Unlike traditional HTA, which primarily informs reimbursement decisions, early HTA is used when the technology and investment decisions can still be changed. Its core purpose is to guide innovators, investors, and developers in prioritising projects, defining target product profiles, and establishing value propositions, ultimately reducing development risks and increasing the likelihood of market adoption^28,29^.

To serve its development-oriented role under conditions of limited evidence, early HTA applies exploratory and iterative analytical approaches. Common methods include headroom analysis, which estimates the maximum price at which a technology could remain value-for-money and provides an early signal of commercial viability, and early decision-analytical modelling, which uses available evidence and explicit assumptions to explore key cost and benefit drivers, assess commercial feasibility, and examine plausible development scenarios^37,38^. Value-of-information analysis is also used to identify evidence gaps where further research would be most valuable, helping to prioritise R&D investments^38^. While these methods are technically similar to those used in traditional HTA, in early HTA they are applied to support strategic R&D decision-making and evidence prioritisation rather than to inform reimbursement decisions.

The differences between early HTA and traditional HTA extend beyond timing to encompass distinct research questions, primary users, evidence sources, and decision-making contexts. Table 1 summarises these key distinctions, highlighting how early HTA is fundamentally oriented toward guiding development and investment, whereas traditional HTA focuses on coverage and reimbursement decisions.

**Table 1:** Differences between traditional HTA and early HTA

	Traditional HTA	Early HTA
Research Question	To facilitate policy decision making: Cost-effective technology? Budget required to roll out the technology? Include in the universal health coverage package? Any organisational, social, legal, ethical, and equity issues?	To facilitate innovation investment and development: Value proposition? Investment in further R&D? Target product profile? Research priority?
Main user	Policymakers	Investors and innovators
Time of Assessment	Later stage when the technology is fully developed^a^	Early stage when the technology and investment decisions can be changed
Perspective	Societal, Payer, Health system, Patient	Technology developer and manufacturer, Industry, Societal, Payer, Health system, Patient
Evidence source	Published literature Real-world data Clinical trials, submitted by the innovators	Published literature Preliminary data Hypothetical assumptions or estimates by the innovators
Funding for evaluation	Public funding Submission fee by the innovators to HTA agencies	Public funding Commission fee from innovators or investors to the early HTA research team
Transparency	Results and decisions are publicly available	Results are often confidential
Decision-making context	Static: Choose the technologies/interventions that are value-for-money compared to the standard care, current practice, and/or golden standard.	Dynamic: Forward looking Consider the potential technologies in the future Current decision can affect the subsequent decisions
Reference case, method, and process guideline, reporting guideline	Many are available for example: ICER’s Reference case^90^ iDSI’s Reference case^91^ Consolidated Health Economic Evaluation Reporting Standards^92^ Guideline from local HTA agencies	Academic literature on methods and frameworks^38,58,93^ Interim guidance or guidance on specific component of early HTA^94^

*HTA* Health Technology Assessment, *ICER* Incremental cost-effectiveness ratio, *iDSI* International decision support initiative, *R&D* Research and Development.

^a^Some HTA agencies will only initiate the traditional HTA after the technology obtains regulatory approval.

## The current landscape of early HTA

HTA agencies and researchers worldwide have begun to realise the importance of early HTA. The National Institute for Health and Care Excellence, United Kingdom, recently established Early Value Assessment (EVA)^39^ to prioritise areas in health and social care, and medical technologies, and completed its first early HTA for medical technology in February 2023^40^. The ASSESS Project in North America was established in 2022 to provide early HTA services to help medical innovators identify their innovations’ commercial potentials to maximise commercial success and market access opportunities^41^. MaRS EXCITE, based in Ontario, Canada, was established in 2011 with the aim to support health technology development through pre-market evaluation^42,43^. In Southeast Asia, the Health Intervention and Technology Assessment Programme Foundation (HITAP) and Saw Swee Hock School of Public Health, National University of Singapore (NUS) co-founded the Medical Innovation Development and Assessment Support (MIDAS) in 2023. MIDAS’ objectives are to support health technology development through early HTA by guiding health innovators and facilitating innovation development to improve health outcomes and increase accessibility to health technologies within the healthcare system^44^. Not until recently that relevant stakeholders and experts reached a consensus on the definition and scope of early HTA as HTA conducted to inform decision on further development, research, or investment of a concept or an emerging health technology^45^. Despite growing interest among researchers, health economists, and policymakers, early HTA remains relatively new to medical innovators. This perspective article aims to raise awareness of early HTA among medical researchers, innovators, and funders and provides a framework for the application of early HTA in a real-world setting by building on existing literature and identifying gaps from existing reviews^19,20,38,46–50^.

The low awareness and misconception of early HTA can leave detrimental and costly consequences for both innovators and patients. Recently, many organisations have started to introduce early HTA into their health technology development pipeline^18,38,51–53^. Early HTA initiatives such as ASSESS, EVA, MIDAS and others have been raising awareness of early HTA as well as utilising early HTA to assist health innovation^18,54,55^. Despite these global and regional efforts, understanding and appreciation of early HTA among medical innovators and clinicians remain limited.

Globally, efforts to raise awareness and build capacity for early HTA are growing. These include specialised training workshops for innovators and clinicians, the integration of early HTA modules into health economics curricula, and the use of innovation competitions to demonstrate its practical application^56,57^. In Thailand and Singapore, for instance, such initiatives have been implemented to familiarise stakeholders with early HTA principles. Furthermore, translating early HTA findings into policy briefs and prioritisation tools has been emphasised as a strategy to bridge research and decision-making^58–62^.

**Fig. 2:**
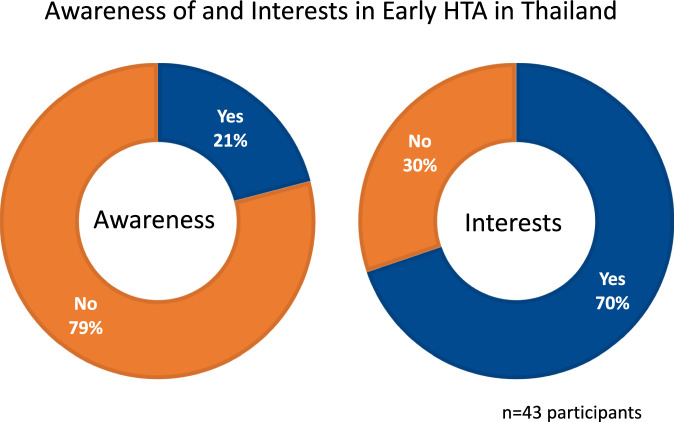
Relatively low awareness of early health technology assessment in Thailand. Survey results based on participants from Mahidol-Oxford Translational Innovation Partnership (MOTIP) networking conference in Bangkok, Thailand, on November 8^th^, 2022, revealed that, when early HTA was first formally introduced to Thai innovators, the awareness of early HTA in Thailand was relatively low but there was high interest in early HTA services for health technology development. Of all participants, 43% has experience in health technology development with 26% expressing that their biggest concern on innovation development was market authorisation, and 38% worried about the cost of conducting early HTA. Note: These survey results are descriptive and were collected for instructional purposes; they are not derived from a formal, statistically powered research study.

**Fig. 3:**
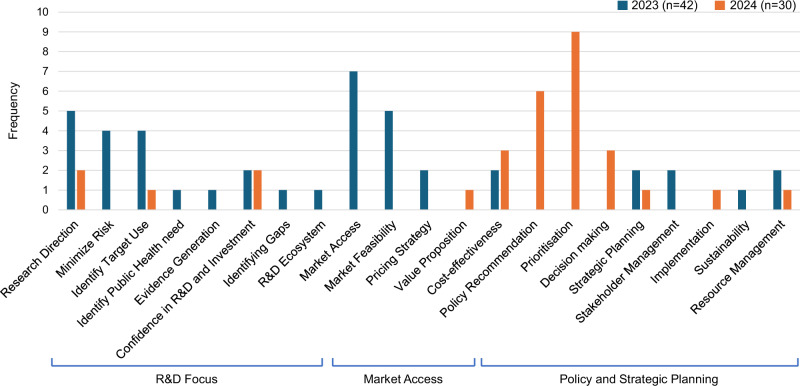
Comparison of opinions of benefit of early HTA among innovators who attended an annual health Economic Evaluation training in Thailand. Survey results based on attendees at an annual Health Economic Evaluation training in 2023 and 2024, hosted by the Health Intervention and Technology Assessment Programme Foundation, revealed a the shift in opinions of the benefit of early HTA on health technology research and development. Trainees included healthcare professionals, policymakers, academics, innovators from public and private sectors, and medical students. Of all the trainees, 42 out of 121 answered the survey in 2023 and 30 out of 33 trainees answered the survey in 2024. Note: These survey results are descriptive and were collected for instructional purposes; they are not derived from a formal, statistically powered research study.

Illustrative feedback from participants in these regional activities suggests that while initial awareness of early HTA may be low, interest is rapidly growing. For example, surveys conducted during related workshops and conferences in Thailand indicated a shift in perception among innovators—from viewing early HTA as a tool for R&D risk minimisation to recognising its role in system-level prioritisation and policy guidance (Figs. 2 and 3). These observations, though descriptive, highlight an evolving understanding of early HTA’s value in the ecosystem.

## When should different stakeholders engage early HTA researchers?

### A product lifecycle and technology readiness level (TRL)

Like other technologies, health technologies begin as ideas from innovators (TRL1) and make their way into the healthcare market and to consumers (TRL9) (Fig. 1). Typically, products and innovations go through five stages: (1) R&D, (2) introduction, (3) growth, (4) maturity and (5) decline^63,64^. Ijzerman et al. (2011) further identified four stages considering a typical health technology: (1) basic research, (2) translational research, (3) clinical research and (4) market access^46^. For medical and health innovators, this translates to TRL when assessing the maturity of innovations and resource allocation (Fig. 1)^65^, and pivotal decisions are made during each step and level in R&D based on the technology’s feasibility and potential profits. This critical phase serves as the incubator for health technology where scientific insight, technical viability, and market potential interplay.

### Basic research

There is little empirical data to assess an innovation’s potential at this stage. Despite invested in medical product development^66^, the benefits and monetary returns of basic biomedical research remain debated. Evaluating the payback of investing in basic research usually relies on historical data which may not be predictive to justify future investment^46,67^. During medical device R&D, basic scientific methods provide quantitative and qualitative evidence that inform practical features for conceptual product designs^68,69^. The most practical conceptual designs will then be selected for the final prototype and implementation^69^. Innovators and early HTA researchers can conduct landscape reviews to identify existing knowledge gaps, assess feasibility, and evaluate whether the necessary methods, technologies, and resources are achievable. Early HTA researchers may elicit information from scientific perspective through expert’s opinions^70^. They can also engage relevant stakeholders and policymakers to help understand the major issues and policy priorities and consult practitioners and patients to identify real-world pain-points. This can help guide investment direction in basic research and prioritise innovations with translational potential.

### Translational research

At this stage, medical innovators focus on their innovation’s impacts on patients and healthcare system, making decisions based on both research and marketing impacts^71^. They apply knowledge from basic research to create new interventions that would directly benefit patients^17,23,72^. Early HTA supports innovators in understanding product impacts and funding potential by gathering evidence on clinical performance and effectiveness to ensure that their prototypes convey value to funders and regulators^73,74^. It defines the desired characteristics of the medical product aimed at a particular disease (target product profile) and identify value propositions to guide health technology development, addressing unmet clinical needs while ensuring safety, effectiveness, and market viability^75^. However, clinical impacts alone are not sufficient for successful market access as medical products should justify additional cost over added outcomes^2,18,76^. Uncertainties in market viability and regulation make predicting value-for-money difficult, highlighting the importance of early EE. Quantitative simulations though modelling can help innovators understand the effects of innovations on the target population, organisation, and society^29,52^. The early cost-effectiveness analysis of an emerging health technology can help steer the implementation of the innovation by both industry and government^18,77^. Early HTA methodologies can help innovators identify the evidence required such as the effectiveness of the emerging technology, inform how to generate such evidence, and set plans and goals for innovators to achieve.

### Clinical research

At clinical research stage, a new health technology is first introduced to patients. Early HTA can identify factors and outcomes that are essential for evaluating both medical and economic aspects of clinical trials and increase efficiency of research. Yet, many early HTA studies in recent medical technology development were conducted post-clinical trials^37,78,79^. The results from these studies cannot be used to inform product development and investment decisions. Ideally, early HTA and early EE should be initiated before an innovation enters its first clinical trial and iteratively updated as evidence emerges during subsequent clinical and feasibility studies, rather than after its introduction to real-world patients^18^. Early HTA researchers can perform economic modelling to determine an innovation’s value-for-money and recommend price adjustment, as well as identify the setting and protocol to maximise its value. As evidence accumulates, key information gaps and uncertainties should be explored to inform further trial design. Here, early HTA allows stakeholders to make strategic decisions about resource allocation, including identifying the most cost-effective interventions to include in further clinical trial design and optimise the clinical trial designs over time to maximise the innovation’s value-for-money, accelerate approval process, and ensure its market viability.

### Market access

At this level, traditional and early HTA’s begin to overlap in terms of scopes and evidence considered (Fig. 1). Ideally, innovators should engage in market access analysis at an early stage when their innovation is still under development. This helps anticipate and address challenges related to efficacy, economic value, and feasibility. Value-based evaluation incorporates cost-utility analysis, linking clinical benefits to payers’ willingness to pay and adopt the technology^46,80^. The goal of early HTA is to gain market insights and economic implications that support R&D-stage decision-making. While early HTA is not intended to be used for health coverage programme development, its findings can guide innovators in prioritising their innovations based on public healthcare priorities. If publicly available, insights from early HTA may later be used to assist topic selection and adapted to assist formal assessments during reimbursement decision making process, including drawing on early HTA findings, defined use case, elements of economic models and parameter inputs. It is primarily a valuable tool to guide innovators in navigating the complex landscape of market access and adoption in healthcare, from conceptualisation to implementation of emerging technologies.

In summary, stakeholders involved in the development of health technologies can engage in early HTA starting from the conceptual of product ideas up to initial clinical study stage. Early HTA essentially helps promote more efficient innovation development and reduce risks and time needed for the implementation of the health technology. The key benefits of early HTA applications across early product development stages include:

1. Reducing Research Waste: Research projects that are unlikely to result in successful translational outcomes can be identified early. This helps in reducing risks and avoiding research waste by preventing the continuation of projects that are less likely to lead to meaningful clinical applications in the real world.
2. Facilitating Collaboration: Early involvement of HTA experts can encourage collaboration between innovators (academic or industry), clinicians, patients, HTA professionals and decision makers. Early HTA activities are often commissioned by the innovators and funders to inform development and investment decisions^18,61^. It provides a structured opportunity for engagement among relevant stakeholders, including decision makers, clinicians and patients, throughout the development in the form of stakeholder consultation and result validation. This interdisciplinary approach fosters a more comprehensive understanding of the innovation’s potential impacts and facilitates smoother translation into clinical practice. By clarifying the potential value and use cases of an innovation at early stages, patients and clinicians can contribute to defining needs, outcomes, and implementation considerations, thus enhancing the relevance and acceptability of the emerging innovation.
3. Aligning regulatory and reimbursement needs: Regulatory and reimbursement decision pathways are often on different timelines with different evidence expectation^81^. Early HTA may help bridge this evidence gap by informing evidence generation that meets both regulatory and payer requirements, thus aligning regulatory and reimbursement processes. For example, an early HTA of a new diagnostic might identify that collecting data on both diagnostic accuracy and downstream treatment costs during a pivotal trial is necessary to satisfy both regulatory (safety/efficacy) and payer (cost-effectiveness) evidence requirements^61^. As such, results from early HTA can help inform the innovators on clinical trial designs and parameter selection to address evidence relevant to regulatory approval while generating evidence to support later formal HTA and reimbursement decisions, including pricing strategy and value proposition^82,83^.

## Misperceptions of early HTA

### Early HTA to identify cost-effective innovation for coverage decisions

A common misperception of early HTA is its only use for reimbursement decisions. While it provides insights into health technologies’ potential values, it is based on limited evidence and value may change as innovations develop. Conclusions on value-for-money derived from early economic modelling should be interpreted with cautions, as these analyses are often preliminary, with high uncertainties, and maybe biased towards favourable outcome^84,85^. Early HTA is a tool to guide stakeholders in understanding the feasibility, cost-effectiveness and value propositions of an emerging health technology during R&D, contrasting with traditional HTA’s focus on reimbursement^20,24^. For example, in this study on soft robotic sock, early HTA results informed the innovators of the price premium of their medical device which would increase the probability of the device being considered cost-effective and recommended the innovators incorporating a sensing system to measure the compliance rate to generate more evidence that would be used for determining the cost-effectiveness of this device in the future^18^. The generated evidence was not intended for reimbursement application but for the innovators to modify the prototype before entering clinical trial phases and gaining market authorisation. Reimbursement decisions typically require robust clinical evidence, long-term outcomes, and budget impact analysis. Early HTA, in contrast, engages stakeholders in the strategic planning and decision-making process, offering a preliminary assessment that informs further R&D investment and market access considerations.

### Early HTA to make realistic prediction of technology impacts

Another misperception of early HTA is that it is assumed to predict definitive outcomes and impacts of an innovation. The R&D of health technologies is an iterative process with constant changes from technical specs to design, and to market strategies^86^. Early HTA is an adaptable tool that can lay frameworks based on uncertainties of R&D due to limited data and ongoing development. As new data and evidence emerge, early HTA adapts its models to gain insights into the product’s viability as the technology progresses.

### Early HTA is the same as early dialogue/scientific advice/awareness

A common misconception is that early HTA is equivalent to early dialogue or scientific advice with regulators and HTA agencies. Early HTA is typically innovator- or funder-focused, and uses exploratory clinical and economic assessments to inform innovation design, value proposition and evidence required during R&D. On the other hand, early dialogue and scientific advice focus on clarifying formal regulatory and reimbursement evidence requirements for innovators, while early awareness entails identifying emerging technologies of interest to assess their potential impacts at system levels^45^. However, in practice, some degree of overlap may occur between early HTA and early dialogue, particularly in the early stages of evidence planning and stakeholder engagement. Early HTA, early dialogue and early awareness can complement each other in supporting innovators.

### Early HTA as a panacea

While early HTA builds its predictive models around high level of uncertainties and limited data, it is not a bypass to overcome the lack of robust data and evidence. It is one of many components of a broader evaluation process that provides strategic approaches to make informed decisions based on the value of an emerging health technology and its impact on the healthcare system^87^. Economic modelling cannot substitute for clinical studies because models rely on assumptions and extrapolations that may not fully capture the complexities and nuances of actual clinical outcomes^88^. It is not a replacement for research to gather concrete evidence through clinical studies and validations for key stakeholders to make informed decisions later, but a predictor of the future outcomes to be used in the decision-making process to ensure the desirable outcomes.

## Challenges and considerations when conducting early HTA

### Time constraints in health technology development

Health technology R&D moves at a fast pace, especially with the increasing pressure for new medical products, thus putting time constraints on conducting an early HTA. This imposes short timeframes on health technology R&D as innovators must balance between delivering quality products and rushing to put their products on the market. In addition, all stakeholders must deal with this time constraint to ensure that the insights gained from early HTA are timely and relevant for guiding effective decision-making in view of the dynamic and competitive landscape of health innovation. Depending on the scope of the early HTA activities, such as horizon scanning, landscape reviews, stakeholder consultations, expert interviews, and modelling, the time required to conduct early HTA may range from a few weeks to approximately 6–8 months^18,61^.

### Confidentiality in data used and assessment

With its many stakeholders in the healthcare sector, confidentiality considerations are crucial when conducting early HTA. As the assessments often involve proprietary information about emerging health technologies, several key confidentiality issues need attention to ensure transparency^89^. Early HTA involves sensitive information such as detailed specifications of the innovation under development. Strict adherence to privacy regulations is essential to protect the interest of the medical innovator and ensure long-term collaboration. A clear and comprehensive collaboration agreement should be established between all stakeholders involved to define the scope of the research itself as well as information sharing to ensure that sensitive data is protected. It is important to recognise that specific confidentiality considerations may vary based on the nature of the health technology of interest, the stakeholders involved, and the regulatory landscape in each country. Unlike HTA, where assessment reports and reimbursement decisions are typically publicly available, results from early HTA may be confidential, used primarily for internal decision-making by the innovators, or partially disclosed in generic terms without detailed information on the specific intervention^59^. Such confidentiality considerations in early HTA may further contribute to low awareness and limited visibility of early HTA activities among stakeholders.

### Ethics application

Ethics application is mandatory for most biomedical research, particularly for research involving patients, sensitive data, and the public. However, the ethical application process can be time consuming, and researchers cannot predict the exact time required for approval. Given the time sensitive nature of innovation development, this may hinder collaboration with the innovators as they may be reluctant to committing to the early HTA research. When designing early HTA research, researchers should consider the benefit of the study and the need of ethics application. Involvement of domain experts, relevant stakeholders, clinicians, and innovators are important to ensure the quality of the work. Given that early HTA is a new area in health research, research ethics committees may misunderstand early HTA projects and apply clinical research standards that may not be relevant in some cases, such as randomisation of samples or sample size calculations. Waiving ethics application may be appropriate for certain aspects such as expert and stakeholder consultations but involving patients may require at least an expedited ethics application. However, the benefit of involving patients at the early innovation stage may not justify the delay of the research due to ethics application. Similarly, when using confidential data, researchers may prefer aggregate-level information over individual-level data as this reduces privacy concerns and may simplify the ethics approval process.

### Management of conflict of interest

One ethical concern for early HTA is the management of conflict of interest. The fact that public HTA bodies could advise private companies in evaluating their health technologies for public reimbursement can lead to conflicts of interest due to the different priorities and objectives of public and private sectors. To address this concern, it is important for early HTA processes to be transparent in disclosing and managing conflicts of interest, while still retaining confidentiality between all parties involved. Establishing clear guidelines and records helps ensure that all early HTA activities are conducted with integrity and prioritise public health interests over potential private influences. This transparency contributes to maintaining trust in the entire HTA process and the credibility of the recommendations provided for healthcare decision-making.

## Conclusions

Health technology R&D involves high levels of uncertainty stemming from human variability, the research process, and costs. Health technology development requires constant changes and refinement in features, designs, properties, effectiveness, and cost. In order to reduce the uncertainty concerning clinical effectiveness, regulation, market access, and return on investment, a more adaptive and flexible assessment like early HTA is essential throughout the medical product lifecycle. Early HTA not only evaluates the current state of technology but also considers its potential for future advancement. It integrates a foresight aspect into the conventional HTA that includes both technical and economic evaluations, resulting in a predictive capacity that enables a more thorough evaluation of a developmental-stage technology’s long-term viability, adaptability, and adoptability in the healthcare system.

### Ethics approval

This work did not require Ethics approval.

## Data Availability

No research data was generated during the current study.
